# Glucagon-Producing Pancreatic Neuroendocrine Tumors (Glucagonomas) are Enriched in Aggressive Neoplasms with ARX and PDX1 Co-expression, *DAXX*/*ATRX* Mutations, and ALT (Alternative Lengthening of Telomeres)

**DOI:** 10.1007/s12022-024-09826-z

**Published:** 2024-09-27

**Authors:** Paola Mattiolo, Michele Bevere, Andrea Mafficini, Anna Vera D. Verschuur, Martina Calicchia, Wenzel M. Hackeng, Michele Simbolo, Salvatore Paiella, Koen M. A. Dreijerink, Luca Landoni, Serena Pedron, Sara Cingarlini, Roberto Salvia, Michele Milella, Rita T. Lawlor, Gerlof D. Valk, Menno R. Vriens, Aldo Scarpa, Lodewijk A. Brosens, Claudio Luchini

**Affiliations:** 1 Department of Diagnostics and Public Health, Section of Pathology, University of Verona, Piazzale L.A. Scuro, 10, 37134 Verona, Italy; 2 ARC-NET Applied Research On Cancer Center, University of Verona, Verona, Italy; 3 Department of Engineering for Innovation Medicine (DIMI), University of Verona, Verona, Italy; 4 Department of Pathology, UMC Utrecht, Utrecht, the Netherlands; 5 Department of General and Pancreatic Surgery, The Pancreas Institute, University and Hospital Trust of Verona, Verona, Italy; 6 Department of Endocrinology, Amsterdam UMC, Amsterdam, the Netherlands; 7 Department of Pathology, UMC Utrecht, Utrecht, the Netherlands; 8 Unit of Oncology, University and Hospital Trust of Verona, Verona, Italy; 9 Department of Endocrine Oncology, UMC Utrecht, Utrecht, the Netherlands; 10 Department of Endocrine Surgery, UMC Utrecht, Utrecht, the Netherlands; 11 Department of Pathology, Radboud University Medical Center, Nijmegen, The Netherlands

**Keywords:** NET, Functioning, Neuroendocrine, Glucagon, MUTYH, DAXX, ATRX, ALT

## Abstract

Glucagonomas are functioning pancreatic neuroendocrine tumors (PanNETs) responsible for glucagonoma syndrome. This study aims to shed light on the clinicopathological and molecular features of these neoplasms. Six patients with glucagonomas were identified. All neoplasms were investigated with immunohistochemistry for neuroendocrine markers (Synaptophysin, Chromogranin-A), ATRX, DAXX, ARX, and PDX1 transcription factors. Fluorescent in situ hybridization (FISH) for assessing alternative lengthening of telomeres (ALT), and next-generation sequencing (NGS) for molecular profiling were performed. All cases were large single masses (mean size of 8.2 cm), with necrolytic migratory erythema as the most common symptom (6/6 cases, 100%). All neoplasms were well-differentiated G1 tumors, except one case that was G2. The tumors consistently showed classic/conventional histomorphology, with solid-trabecular and nested architecture. Lymphatic and vascular invasion (6/6, 100%), perineural infiltration (4/6, 66.6%), and nodal metastasis (4/6, 66.6%) were frequently observed. Transcription factors expression showed strong ARX expression in all tumors, and PDX1 expression in 5/6 cases (83.3%), indicating co-occurring alpha- and beta-cell differentiation. NGS showed recurrent somatic *MEN1* and *ATRX/DAXX* biallelic inactivation. Cases with *ATRX* or *DAXX* mutations also showed matched loss of ATRX or DAXX protein expression and ALT. One case harbored somatic *MUTYH* inactivation and showed a high tumor mutational burden (TMB, 41.0 mut/Mb). During follow-up, one patient died of the disease, and four patients developed distant metastasis. Pancreatic glucagonomas are distinct PanNETs with specific clinicopathological and molecular features, including histological aspects of biological aggressiveness, co-occurring alpha- and beta-cell differentiation, *MEN1* and *DAXX*/*ATRX* mutations enrichment, and the possible presence of high-TMB as an actionable marker.

**Supplementary Information:**

The online version contains supplementary material available at 10.1007/s12022-024-09826-z.

## Introduction

Functioning pancreatic neuroendocrine tumors (PanNETs) represent up to 15–20% of all PanNETs and are classified based on the secreted peptide [1–4]. One subtype of functioning PanNETs is represented by glucagon-producing PanNETs (glucagonomas). They are composed of neoplastic cells that produce glucagon and preproglucagon-derived peptide [1–4]. The uncontrolled secretion of these molecules causes glucagonoma syndrome, whose typical clinical manifestations include necrolytic migratory erythema, deep vein thrombosis, new-onset diabetes mellitus, weight loss, and depression [1, 5]. The diagnosis of glucagonoma syndrome is based on the combination of such symptoms and elevated glucagon levels in the plasma [1, 4–6].

Usually, glucagonomas are represented by solitary and large tumor masses with pushing borders. Histologically, they are hypercellular and well-differentiated PanNETs [1]. At immunohistochemistry (IHC), tumor cells are positive for keratin, neuroendocrine markers including synaptophysin and chromogranin-A, and glucagon [1]. Notably, PanNETs showing positivity for glucagon at IHC but lacking clinical manifestations do not satisfy the diagnostic criteria and are thus defined as non-functioning glucagon-producing PanNETs or alpha-cell PanNETs. To date, knowledge of the histomolecular features of glucagonoma is limited. Still, comprehending these aspects may have important clinical implications, as already demonstrated for non-functioning and other subtypes of functioning PanNETs, such as insulinomas [7, 8].

This study aims to define the histomorphology, alpha- and beta-cell differentiation, and molecular features of this rare functioning subtype of PanNETs by investigating a cohort of patients with pancreatic glucagon-producing tumors.

## Materials and Methods

All glucagonomas diagnosed at three different Italian and Dutch Institutions in the last 25 years and with available slides and tissue blocks were identified. The study has been approved by the local ethics committee (no.17/881, 52,070/CE, 21/507). Medical records and electronic databases were reviewed to obtain clinical and pathological data. All neoplasms were investigated with immunohistochemistry (IHC), fluorescent in situ hybridization (FISH) for assessing alternative lengthening of telomeres (ALT), and next-generation sequencing (NGS) for molecular profiling.

### Immunohistochemistry

IHC was performed as previously described [8–10], with the following antibodies and according to the manufacturer’s instructions: cytokeratin AE1/AE3 (clone: AE1-AE3, 1:100 dilution, source/country: Novocastra/UK), Chromogranin-A (DAK-A3, 1:2500, Dako/Germany), Synaptophysin (27G12, prediluted, Novocastra), Ki-67 (MIB1, 1:100, Dako), Glucagon (mAb/rabbit, 1:15,000, Abcam/UK), Insulin (polyclonal/guineapig, 1:200, Dako), Somatostatin (polyclonal/rabbit, 1:1000, Dako), DAXX (polyclonal/rabbit, 1:200, Sigma/USA), ATRX (AX1, 1:200, Dianova/USA), ARX (11F6.2, 1:50, Millipore/USA), and PDX1 (EPR3358(2), 1:50, Abcam/UK).

### *Fluorescent *in Situ* Hybridization*

Telomere-specific FISH for assessing ALT was performed as previously described [11] and using a fluorescently tagged telomeric-C PNA probe. Scoring for ALT was performed by assessing at least 250 nuclei for each case. Using previously described criteria [9], ALT-positive cases were defined by the presence of large, ultrabright intranuclear foci consistent with telomere FISH signals in at least 1% of tumor nuclei and the total signal intensity for individual foci > tenfold than telomere signals from stromal cells.

### Next-Generation Sequencing

NGS was performed using the SureSelectXT HS CD Glasgow Cancer Core assay (www.agilent.com), hereinafter referred to as CORE, as described elsewhere [12, 13]. Briefly, the panel spans 1.8 megabases (Mb) of the genome and interrogates 174 genes for somatic mutations, copy number alterations, and structural rearrangements; a detailed list of targeted genes is reported in Supplementary Table 1. Variants were classified following the five-tier classification system recommended by the joint consensus of the American College of Medical Genetics and Genomics and the Association for Molecular Pathology (ACMG/AMP) [14].

### Assessment of Possible Germline Variants

In those cases where NGS detected potential germline variations, a specific Sanger sequencing was performed on matched normal tissue (the spleen for cases #1, #2, #3, and #4; the duodenum for cases #5 and #6; blood not tested).

## Results

### Clinicopathological Assessment

Six cases of clinically functional glucagon-producing neuroendocrine tumors of the pancreas were identified (for an estimated prevalence of glucagonomas among all resected functioning and non-functioning PanNETs in the institutions: 0.5%). All tumors were represented by a single mass and with a mean size of 8.2 cm (range: 3.5–14). The clinicopathological features of all cases have been summarized in Table 1. Briefly, there were four male and two female patients, with a mean age of 62.5 years. Most cases involved the pancreatic tail (4/6, 66.6%). Clinically, all patients had necrolytic migratory erythema, and all but one case experienced recently-onset diabetes mellitus.

**Table 1:** Summarizing table of the most relevant clinicopathological parameters and mutations of all cases

ID case	Sex	Site	Size (cm)	Ki-67, grade	LI/VI	PNI	Hist, %f	pTNM	Clin MN	Gluc BL	DSS (m)	RFS(m)	TMB	Gene	Mutations^	Class	ALT
1	M	T	14.0	1%, G1	Yes/Yes	Yes	CL, 20%	T3N0M0	Derm, DM	700	> 60	BM, LM (24)	41.0	*MUTYH*	p.A253E	4	No
2	F	T	3.5	2%, G1	Yes/Yes	No	CL calc, 30%	T2N1M0	Derm	350	> 60	PGM (24)	3.8	*MEN1* *DAXX*	p.I85fs*33 c.1715 + 1G > A	5 5	Yes
3	M	T	8.0	2%, G1	Yes/Yes	Yes	CL, 30%	T2N1M0	Derm, DM	29	> 60	> 60	2.7	*MEN1* *ATRX*	p.W423* p.L2228F	5 4	Yes
4	M	T	6.0	1%, G1	Yes/Yes	Yes	CL, 30%	T3N1M1	Derm, DM	514	36	NA	5.9	*MEN1*	p.H272fs*44	4	No
5	M	H	10.0	8%, G2	Yes/Yes	Yes	CL, 10%	T3N1M0	Derm, DM	374	> 60	LM (44)	3.8	*CDKN1A* *DAXX*	p.R83fs*4 c.814 + 3A > C	4 4	Yes
6	F	H	7.5	1%, G1	Yes/Yes	No	CL, 10%	T3N0M0	Derm, DM	204	> 60	LM (228)	3.0	*DAXX*	p.R231*	4	Yes

*LI* lymphatic invasion, *VI* vascular invasion, *PNI* perineural invasion, *Hist* histological pattern, *%f* percentage of fibrosis/stromal component in the tumor mass, *calc* presence of dystrophic calcifications, *pTNM* pathological TNM at the time of surgical resection, *Clin MN* clinical manifestations, *Gluc BL* pre-operative glucagon blood level (pg/ml), *DSS* disease-specific survival (expressed in months from the surgical resection), *RFS* relapse-free survival (expressed in months from the surgical resection), *TMB* tumor mutational burden (mutations/Mb), *ALT* alternative lengthening of telomeres, *M* male patient, *T* pancreatic tail, *CL* classic/conventional histology, *Derm* dermatosis (necrolytic migratory erythema), *DM* diabetes mellitus, *BM* bone metastasis, *LM* liver metastasis, *F* female patient, *PGM* para-gastric metastasis, *H* pancreatic head, *NA* not applicable

Notes: **^**mutations are classified as per American College of Medical Genetics and Genomics and the Association for Molecular Pathology (ACMG/AMP) recommendations

**Fig. 1:**
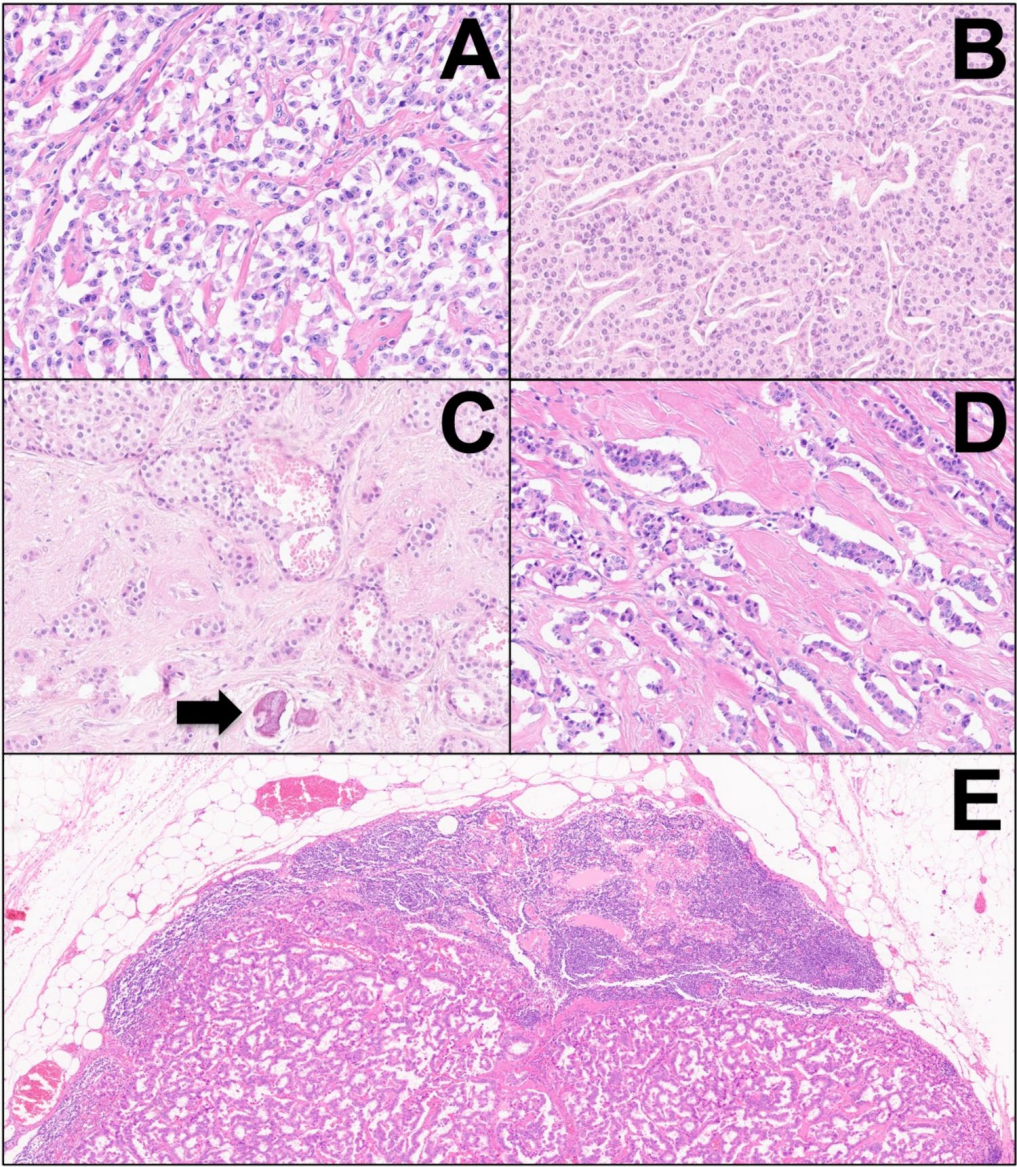
Highly representative microscopic images, stained with hematoxylin–eosin, of the histomorphology of the cases in the current series. All tumors show hypercellular areas with classic/conventional architecture and solid-trabecular features (**A**, **B**; A: 20 × original magnification, B: 20 ×). Areas of hyalinized fibrosis were also detected, representing up to 30% of the tumor mass (**C**, **D**; C:10 × , D: 20 ×). In one case (case #2), dystrophic calcifications were also present (black arrow in C). Nodal metastases were common and detected in 4 cases (**E**, 4 ×)

Histologically, all neoplasms were classified as well-differentiated pancreatic neuroendocrine tumors. Tumor grading was G1 for all neoplasms, except one case that was G2 (Ki-67: 8%). Lymphatic invasion (6/6, 100%), vascular invasion (6/6, 100%), and perineural infiltration (4/6, 66.6%) were common. Tumor morphology was always classic/conventional, with solid-trabecular and nested architecture (Fig. 1). There was a variable amount of hyalinized fibrosis within the tumor mass, ranging from 10 to 30% of the neoplastic volume; one case (case #2) showed the presence of dystrophic calcifications but without the formation of psammoma bodies (Fig. 1). Tumor cells were small-to-medium sized, with finely dispersed chromatin. Nodal metastases were detected in most cases (4/6, 66.6%), and one case also showed a liver metastasis at the time of surgical intervention.

### IHC and FISH

**Fig. 2:**
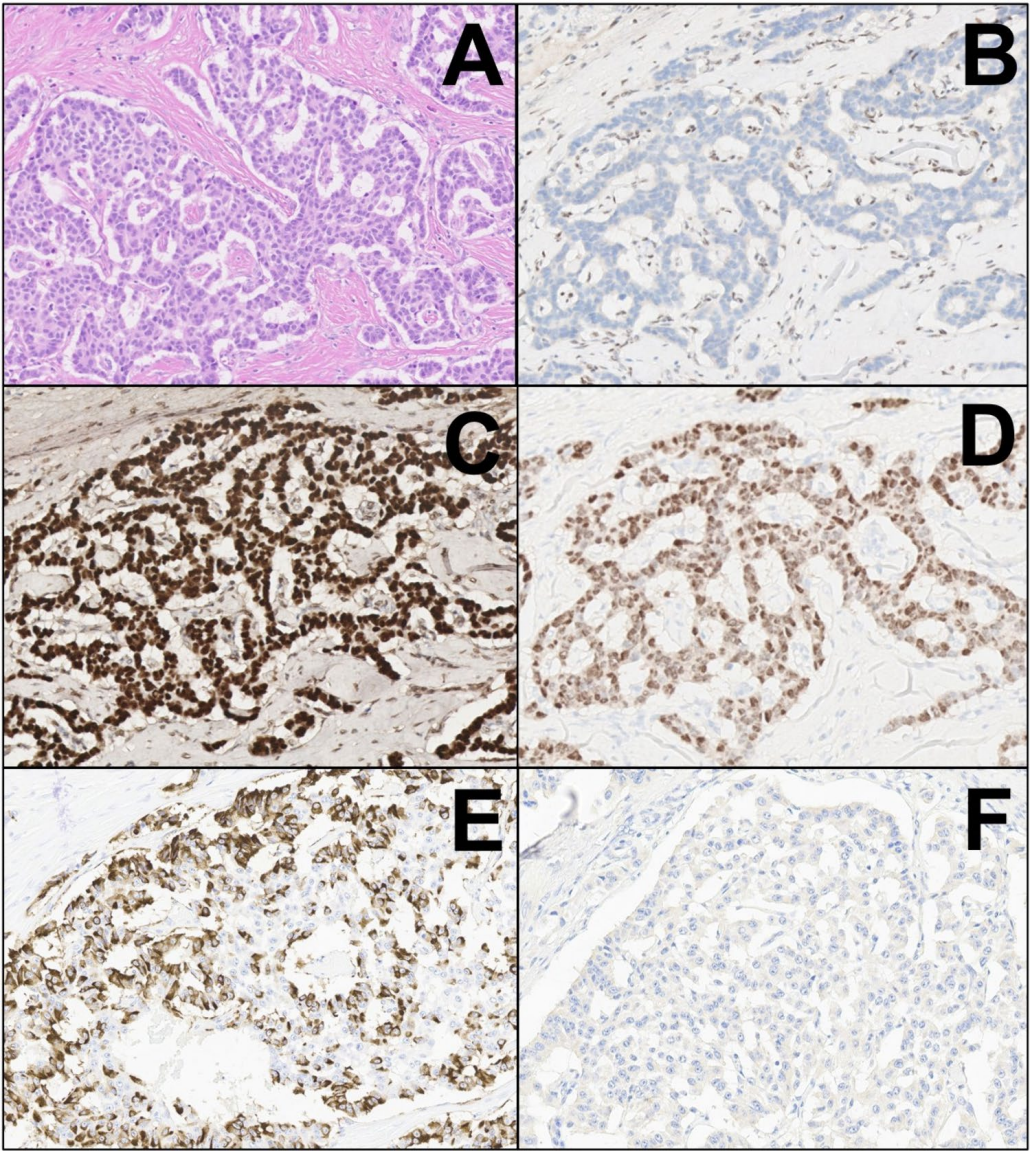
Highly-representative images of some of the most relevant immunohistochemical analyses in the current series (original magnification 20 ×). After showing a classic example of tumor morphology (**A**, hematoxylin–eosin), we showed representative images of the following markers along with their expression pattern: ATRX (loss of expression, **B**), ARX (strong expression, **C**), PDX1 (intermediate expression, **D**), glucagon (strong expression, **E**), and somatostatin (negative, F)

All tumors were positive for cytokeratin AE1/AE3, Chromogranin-A, Synaptophysin, and Glucagon and were negative for Insulin and for Somatostatin (Fig. 2). All tumors were entirely and strongly positive for alpha cell marker ARX; remarkably, 5/6 tumors also showed expression of beta cell marker PDX1, although to a lesser extent than ARX (Fig. 2). Details on the expression patterns of hormones and transcription factors are provided in Supplementary Table 2. DAXX immunostaining showed loss of expression in 3 cases, and ATRX was lost in one case (Fig. 2). FISH documented the presence of ALT in the same four cases that showed loss of ATRX or DAXX expression.

### Molecular Analysis

Pathogenic and likely pathogenic gene mutations are summarized in Table 1. Chromosomal alterations have been summarized in Table 2. Variants of uncertain significance (VUS) have been summarized in Supplementary Table 3.

**Table 2:** Summary of chromosomal alterations in the study’s cohort

ID case	Chromosomal CNV	
	**LOH**	**Gain**
1	chr1p35.1-p13.1, chr1q25.3-q31.3, chr3p26.3-p22.3, chr3p21.31-p21.1, chr6q, chr10q22.2-q26.3, chr11, chr18q12.1-q23, chr21	chr7q22.1-q36.3
2	chr1p, chr1q, chr2, chr3, chr6, chr8, chr10, chr11, chr12, chr15p13-q25.2, chr16, chr21, chr22, chrX	chr7, chr9, chr14, chr15q26.1-q26.3, chr17, chr18, chr19, chr20
3	chr2p, chr6, chr9, chr10, chr11, chr15q15.1-q21.2, chr16, chr22	chr2q, chr4, chr5p, chr5q, chr7, chr8, chr12, chr13, chr14, chr15p13-q14, chr15q21.1-q26.3, chr17, chr18p, chr18q21.33-q22.3, chr19, chr20
4	chr1, chr2, chr3, chr6, chr8, chr10, chr11, chr16, chr18, chr21, chr22	
5	chr1, chr2, chr3, chr6, 8, chr9p24.3-p21.3, chr10, chr11, chr12q21.2-q24.33, chr15, chr16, chr21, chr22	
6	chr1, chr2, chr3, chr5, chr6, chr8, chr10, chr11, chr15, chr16, chr21, chr22	

*CNV* copy number variation, *LOH* loss of heterozygosity, *chr* chromosome

One case was characterized by the presence of a somatic mutation of *MUTYH*, coupled with loss of heterozygosity (LOH) of the region of chromosome 1 containing the gene. The somatic nature of *MUTYH* variation was confirmed with Sanger sequencing of matched normal tissue, where such alteration was not found. This case showed a high tumor mutational burden (TMB, 41.0 mut/Mb). The detected *MUTYH* variation (p.A253E) is in a mutational hot spot and well-established functional domain (i.e., the Endonuclease ENDO3c domain). The high-TMB of this case suggests damage in DNA repair machinery and is consistent with *MUTYH* inactivation. Thus, we performed a mutational signature analysis using the MuSiCa software [15] coupled with the Cosmic human cancer v2 signature database as a reference (https://cancer.sanger.ac.uk/signatures, last access 06/22/2024). The results showed a significant contribution (69.1%) of signature SBS18, which has been associated with defective base excision repair due to *MUTYH* mutations. None of the other tumors showed high-TMB.

Regarding recurrent alterations, *MEN1* was mutated in three cases. In all these cases, *MEN1* mutations were somatic events (confirmed with Sanger sequencing of matched normal tissue) and were always coupled with LOH of the region of chromosome 11 containing the gene. *DAXX* was mutated in three cases and *ATRX* was mutated in another case. These same cases showed corresponding loss of DAXX/ATRX expression at IHC and ALT at FISH. There were also recurrent chromosomal alterations, with the most common represented by LOH involving chromosomes 6, 10, and 11 (6/6 cases, 100%) (Table 2).

### Survival Status

Regarding disease-specific survival, one patient died of the disease during follow-up (mean follow-up period of 94 months). This patient had a liver metastasis at the time of surgical resection (surgery for palliative intent). Furthermore, in a very widely different interval (ranging from 24 to 228 months after surgical resection), four patients developed distant metastasis during follow-up, with the majority of those cases (3/4, 75%) involving the liver.

## Discussion

This study analyzed a cohort of six patients with clinically functional neuroendocrine glucagon-producing neoplasms of the pancreas. All cases were represented by large single masses, with a mean size of 8.2 cm (range: 3.5–14), and arising more often in the pancreatic tail. The most common symptoms were necrolytic migratory erythema and recently onset diabetes mellitus, in line with data from the literature [6]. All neoplasms were well-differentiated G1 tumors, except one case that was G2. Tumor morphology was always classic/conventional, with solid-trabecular and nested architecture. The molecular profile showed recurrent somatic *MEN1* variations coupled with LOH (3 cases) and/or *ATRX/DAXX* mutations (4 cases). Those cases with *ATRX/DAXX* mutations also showed ALT at FISH. Another case was characterized by somatic *MUTYH* mutation and corresponding LOH, showing high-TMB (41.0 mut/Mb). During the follow-up, one patient died of the disease, and four other patients developed distant metastasis.

A critical insight from this study comes from analyzing the clinical behavior of these tumors. Even though they were well-differentiated tumors with classic/conventional features and a low proliferation index at histopathology, they showed morphological aspects of biological aggressiveness. These included consistent lymphatic invasion and vascular invasion, always present, and frequent perineural infiltration and nodal metastases. Therefore, it is not surprising to detect recurrent *ATRX/DAXX* mutations and ALT, which are factors known to correlate with worse prognosis and distant metastasis in functioning (insulinomas) as well as in non-functioning PanNETs [8, 11]. Notably, a recently published case report also described a pancreatic glucagonoma with biallelic inactivation of *DAXX* [16].

Regarding tumor histology, we showed that all tumors in our series showed classic/conventional features. Such aspects demonstrated that glucagon-producing PanNETs did not harbor a distinctive histomorphology from NF-PanNETs. There was also a variable amount of hyalinized stroma within the tumor mass, and one case showed, in this background, the presence of dystrophic calcifications. However, they are not similar to the psammoma bodies commonly found in somatostatinomas [17]. Interestingly, a previous study showed that non-syndromic but glucagon-immunoreactive PanNETs displayed a cystic appearance in a non-negligible proportion of cases [18]. However, all tumors in our series were solid, and this finding further highlights the differences between non-syndromic glucagon-immunoreactive vs. syndromic glucagon-producing PanNETs.

Notably, tumors in this case series were also quite large at the time of diagnosis, with a mean value of 8.2 cm (range 3.5–14). These data are in line with the current literature, which have pointed out that glucagonomas are usually large masses, with an average size around 5 cm [19]. Similarly, large insulinomas are more often aggressive and the late symptomatology usually observed in those cases suggests relatively low or late-acquired insulin production. Of interest, whereas typical small insulinomas show strong PDX1 expression but no ARX expression (as seen in normal beta-cells), aggressive insulinomas also express ARX (lacking in normal beta-cells, but present in alpha- or gamma-cells) [20, 21]. This strongly suggests distinct endocrine (de)differentiation in aggressive insulinomas versus small and indolent insulinomas, with similarities of aggressive insulinomas to non-functional PanNETs which often co-express ARX/PDX1 [7]. Likewise, the fact that all glucagonomas in this series were large and clinically aggressive, showed *ATRX*/*DAXX* mutations in 4/6 cases, and showed unexpected co-expression of non-alpha-cell marker PDX1 in 5/6 cases strongly suggests (epi)genetic similarities with non-functional PanNETs as well as with aggressive insulinomas [8].

Another significant finding is the first description of an actionable alteration in pancreatic glucagonomas. Specifically, one case showed high-TMB resulting from biallelic somatic inactivation of *MUTYH*, which was further validated through the analysis of molecular signature. As observed in this case, tumors with this type of alteration typically show high-TMB [22], which is already an approved target for immunotherapy [23]. Recently, *MUTYH* alterations have been described as a rare genetic driver of well-differentiated PanNETs [23, 24]. Of note, this is the first report of this type of alteration in the context of functioning PanNETs, suggesting the potential of assessing this molecular target in such neoplasms.

This study had some limitations. First, the sample size was relatively small; however, due to their rarity, this case series still represents the largest cohort of molecularly-characterized pancreatic glucagonomas in the literature. Moreover, we did not perform whole-genome sequencing. Thus, it is possible that some potential genetic drivers have not been detected. Still, the adopted CORE panel was conceived to detect the vast majority of the clinically useful molecular alterations in solid epithelial neoplasms.

Overall, pancreatic glucagonomas are distinct neuroendocrine tumors with specific clinical symptomatology and oncologically aggressive aspects but with (epi)genetic features similar to non-functional PanNETs and aggressive insulinomas, including features of alpha- and beta-cell differentiation. Such neoplasms are also enriched in *MEN1* and *ATRX*/*DAXX* mutations and can harbor high-TMB as an actionable target. Their recognition is fundamental for clinical/prognostic implications and precision oncology.

## Supplementary Information

Below is the link to the electronic supplementary material.

Supplementary file1 (PDF 43 KB)

Supplementary file2 (DOCX 14 KB)

Supplementary file3 (DOCX 16 KB)

## Data Availability

No datasets were generated or analysed during the current study.
