# Supplementary material for: Glucagon-Producing Pancreatic Neuroendocrine Tumors (Glucagonomas) are Enriched in Aggressive Neoplasms with ARX and PDX1 Co-expression, DAXX/ATRX Mutations, and ALT (Alternative Lengthening of Telomeres)
Source: Endocr Pathol. 2024 Sep 27;35(4):354–61. doi: 10.1007/s12022-024-09826-z (PMC11659356; doi:10.1007/s12022-024-09826-z)
Supplement: Supplementary file 2 — Supplementary file2 (DOCX 14 KB) [file 12022_2024_9826_MOESM2_ESM.docx]

**Supplementary Table 2**. Expression pattern (whole slide - % of positivity and intensity) of transcription factors and hormones tested in the current study with immunohistochemistry.

| **ID case** | **ARX** | **PDX1** | **Glucagon** | **Insulin** | **Somatostatin** |
| --- | --- | --- | --- | --- | --- |
| 1 | 100% SE | 0% | 90% SE | 0% | 0% |
| 2 | 100% SE | 50% IE | 100% SE | 0% | 0% |
| 3 | 100% SE | 70% IE | 80% SE | 0% | 0% |
| 4 | 100% SE | 20% WE | 70% SE | 0% | 0% |
| 5 | 100% SE | 50% WE | 60% IE | 0% | 0% |
| 6 | 100% SE | 10% WE | 90% SE | 0% | 0% |

**Note**: the evaluation followed an already established methodology (see references 8 and 11 of the current study). **Abbreviations**: SE: strong expression; IE: intermediate expression; WE: weak expression.
