# Supplementary material for: Glucagon-Producing Pancreatic Neuroendocrine Tumors (Glucagonomas) are Enriched in Aggressive Neoplasms with ARX and PDX1 Co-expression, DAXX/ATRX Mutations, and ALT (Alternative Lengthening of Telomeres)
Source: Endocr Pathol. 2024 Sep 27;35(4):354–61. doi: 10.1007/s12022-024-09826-z (PMC11659356; doi:10.1007/s12022-024-09826-z)
Supplement: Supplementary file 3 — Supplementary file3 (DOCX 16 KB) [file 12022_2024_9826_MOESM3_ESM.docx]

**Supplementary Table 3**. Summary of variants of uncertain significance in the study’s cohort.

| **ID case** | **Variant of uncertain significance (VUS)** | | | | |
| --- | --- | --- | --- | --- | --- |
|  | **Gene** | **Variation** | **Mutation type** | **Frequency (%)** | **Class** |
| 1 | *EGFR* | p.Q581K | Substitution - missense | 43 | 3 |
|  | *FBXW7* | p.G571W | Substitution - missense | 51 | 3 |
|  | *KMT2A* | p.P1705H | Substitution - missense | 14 | 3 |
|  | *MET* | p.K846N | Substitution - missense | 27 | 3 |
|  | *MTOR* | p.K425N | Substitution - missense | 32 | 3 |
|  | *NOTCH1* | p.T1844N | Substitution - missense | 42 | 3 |
|  | *NOTCH4* | p.G1058V | Substitution - missense | 48 | 3 |
|  | *NF1* | p.A264S | Substitution - missense | 46 | 3 |
|  | *NOTCH1* | p.C857* | Substitution – stop-gain | 47 | 3 |
|  | *NOTCH1* | p.C1549* | Substitution – stop-gain | 40 | 3 |
|  | *PMS2* | p.C252F | Substitution - missense | 29 | 3 |
|  | *POLE* | p.E1240K | Substitution - missense | 51 | 3 |
|  | *POLE* | p.E1749D | Substitution - missense | 48 | 3 |
|  | *AMER1* | p.H269N | Substitution - missense | 78 | 3 |
|  | *JAK1* | p.H510N | Substitution - missense | 82 | 3 |
|  | *SF3B1* | p.M376I | Substitution - missense | 30 | 3 |
| 2 | *MEN1* | p.S84P | Substitution - missense | 5 | 3 |
| 3 | *FGFR4* | p.C101* | Substitution – stop-gain | 46 | 3 |
|  | *KMT2A* | p.A1140T | Substitution - missense | 94 | 3 |
|  | *POLE* | p.R1077H | Substitution - missense | 46 | 3 |
|  | *ROS1* | p.P207L | Substitution - missense | 10 | 3 |
| 4 | *ARID1A* | p.G889A | Substitution - missense | 67 | 3 |
|  | *DAXX* | p.D256Y | Substitution - missense | 37 | 3 |
| 5 | *EGFR* | p.S511Y | Substitution - missense | 47 | 3 |
| 6 | *ALK* | p.E1526K | Substitution - missense | 88 | 3 |
